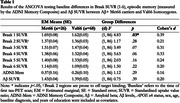# Associations between BDNF Val66Met and tau‐PET and episodic memory performance in sporadic AD

**DOI:** 10.1002/alz.091947

**Published:** 2025-01-09

**Authors:** Diny Thomson, Emily Rosenich, Paul Maruff, Yen Ying Lim, the Alzheimer’s Disease Neuroimaging Initiative

**Affiliations:** ^1^ Turner Institute for Brain and Mental Health, School of Psychological Sciences, Monash University, Melbourne, VIC Australia; ^2^ Cogstate Ltd., Melbourne, VIC Australia

## Abstract

**Background:**

In the presence of abnormally high amyloid (Aβ+), carriage of the brain‐derived neurotrophic factor (BDNF) Val66Met polymorphism Met allele (Met66) is associated with faster clinical disease progression, greater neuronal loss and faster rate of CSF total‐tau and p‐tau_181_ compared to matched Val66 Aβ+ homozygotes. Aβ levels are unaffected by carriage of Met66. This suggests reduced neurotrophic support may accelerate Aβ‐related neuronal dysfunction and cognitive decline. We aimed to clarify the role of BDNF Val66Met in moderating region‐specific neurofibrillary tangle (NFT) formation in the brain in sporadic Alzheimer’s disease (AD).

**Method:**

Data from 26 Aβ+ older adult Met66 carriers and 68 Val66 homozygotes enrolled in the Alzheimer’s Disease Neuroimaging Initiative (ADNI) were included in the analysis if they had at least one tau‐PET scan, Aβ‐PET scan, and neuropsychological assessment available. Participants were not excluded based on disease stage. ANCOVAs were conducted to determine, cross‐sectionally, whether levels of tau‐PET tracer retention at each Braak stage, episodic memory performance (measured on ADNI’s memory composite), and Aβ levels differed between Met66 carriers and Val66 homozygotes at the time of their first tau‐PET scan. Aβ levels, APOE ε4 status, sex, age, baseline diagnosis, and years of education were included as covariates.

**Result:**

Aβ+ Met66 carriers showed significantly greater tracer retention in the entorhinal cortex (i.e., Braak 1) compared to Val66 homozygotes (p=.03), with the magnitude of this difference moderate (d=0.39; Table 1). There were no significant differences between Met66 carriers and Val66 homozygotes in tracer retention across all other Braak stages, episodic memory performance or Aβ levels (Table 1).

**Conclusion:**

The greater tau‐PET tracer retention in the entorhinal cortex in Met66 carriers may reflect their greater NFT pathology relative to Val66 homozygotes during early stages of AD. This is consistent with previous observations of higher levels of CSF total‐tau and p‐tau_181_ in Aβ+ Met66 carriers, both in sporadic AD and autosomal dominant AD. Together, these findings support the hypothesis suggesting that loss of neurotrophic support, associated with Met66 carriage, may confer greater vulnerability to Aβ‐related neurodegeneration compared to Val66 homozygosity.